# Atypical Presentation of Diverticulitis in a Young Hispanic Male: A Case Report

**DOI:** 10.7759/cureus.37511

**Published:** 2023-04-12

**Authors:** Sara Arfan, Ameya Nair, Timothy Stear

**Affiliations:** 1 Department of General Surgery, Windsor University School of Medicine, Cayon, KNA; 2 Department of General Surgery, Saint James School of Medicine St. Vincent, Arnos Vale, VCT; 3 Department of General Surgery, Community First Medical Center, Chicago, USA

**Keywords:** cost burden, diverticulosis, complicated diverticulitis, colovesical fistula, acute diverticulitis

## Abstract

Diverticulitis is a significant contributor to the number of hospital admissions and healthcare costs in Western societies. The authors present the case of an otherwise healthy 33-year-old Hispanic male presenting to the emergency department with complaints of abdominal pain, pneumaturia, and hematochezia. The patient had no underlying risk factors, substantial prior medical history, or typical symptoms of diverticulitis. He was diagnosed with acute diverticulitis with a presumed colovesical fistula. The unique clinical presentation and the intraoperative findings are discussed. The objective of this case report is to make clinicians aware of atypical presentations of acute diverticulitis and guide the appropriate diagnostic workup for young Hispanic males presenting to the emergency departments with complaints of abdominal pain.

## Introduction

Diverticulitis is the inflammation of a colonic false lumen diverticulum. It remains the third most common illness requiring hospitalization, with a reported 27% increase in emergency room visits between 2006 and 2013 in the United States [1,2]. It poses a significant healthcare cost burden in the US with 2.6 million outpatient visits and 200,000 inpatient admissions per year [3,4,5].

Historically, it was believed that diverticulitis predominantly affected men over the age of 60. An increased incidence has recently been reported in individuals under 40 [6]. Hence, the emphasis has switched in recent years to investigating its consequences in the latter group [7]. Some studies have debated and compared the virulence, recurrence rates, and future complications between the two groups [8]. In contrast, others have reported severe clinical symptoms, frequent surgical interventions, and higher morbidity and mortality in individuals under 40 [6]. There is also a debate on the management protocol between the two age groups that are representative of their disease course and outcomes [6,9]. To the best of our knowledge, differences in disease presentation among these two age groups have been vastly underreported, which poses a risk of misdiagnosis, additional testing, and ultimately a higher cost burden on healthcare resources [10]. Our goal is to make clinicians aware of the atypical clinical presentation of acute diverticulitis in the younger demographic and recommend judicious use of imaging, which would decrease the cost burden on healthcare resources. 

Risk factors for acute diverticulitis include advanced age, smoking, obesity (BMI > 30 kg/m^2^), constipation, and low fiber intake [6]. Classical symptoms include fever, crampy abdominal pain, tenderness in the left lower quadrant (LLQ), a change in bowel habits, and blood in the stool. Pneumaturia and fecaluria are often present as signs of the development of a colovesical fistula (CVF) [3]. In ‘complicated’ diverticulitis, in addition to CVF, patients may also develop an abscess or a perforation.

We report an atypical presentation of acute diverticulitis in a healthy 33-year-old Hispanic male with an absence of classical risk factors and no prior medical, surgical, or family history. We also highlight his intraoperative findings.

## Case presentation

A 33-year-old Hispanic male presented to the emergency department complaining of localized mid-epigastric abdominal pain, which was associated with nausea and an episode of nonbloody, nonbilious emesis. The onset of symptoms started 24 hours prior to the presentation while the patient was lifting heavy boxes at work. His pain progressed overnight which prompted his presentation to the emergency department. Upon evaluation, he reported blood in his stool, tea-colored urine, fecaluria, and visible air bubbles during urination, indicative of pneumaturia. He denied any changes in bowel movements.

The patient reported no significant past medical or surgical history and denied similar episodes of abdominal pain in the past. He has no known family history of colorectal disease or cancer. The patient is a nonsmoker and does not consume alcohol or use any recreational or illicit drugs. He has no history of previously prescribed medications or known drug allergies. 

On admission, he was diaphoretic, afebrile, tachycardic, and hypertensive. Hemodynamic parameters on admission included a temperature of 98.6 F, a pulse of 110/min, a respiratory rate of 20/min, blood pressure of 156/76, and oxygen saturation of 94% on room air. His body mass index (BMI) was 32.8 kg/m^2^. A review of systems was positive for abdominal pain, blood in the stool, and dark urine but otherwise negative. On physical examination, the abdomen was soft with severe focal epigastric tenderness and no tenderness in the lower or lateral quadrants. There was no rebound tenderness, guarding, or rigidity. Bowel sounds were decreased in all quadrants. 

Laboratory evaluation demonstrated an elevated serum lactate of 3.2 mmol/L (reference range: 0.5 - 2.2 mmol/L) and an elevated white blood cell count of 22.8 µL (reference range: 4.5 to 11.0 × 109/L). Urinalysis revealed a cloudy appearance with the presence of large amounts of blood, proteinuria, nitrates, and many bacteria (Table 1). 

**Table 1:** Patient laboratory values on presentation and their interpretation CBC: Complete blood count; RBCs: red blood cells

Laboratory Investigation	Patient Laboratory Result	Reference Range	Interpretation
CBC			
Lactate	3.2 mmol/L	0.5 - 2.0 mmol/L	Elevated
White blood cell count	22.8 k/mm cu	4.5 - 11.0 x 10^9^/L	Elevated
Anion gap	12.0 mmol/L	3.6 - 11.0 mmol/L	Elevated
CO_2_	24.0 mmol/L	21 -31 mmol/L	Normal
Glucose	110 mg/dL	70 - 99 mg/dL	Elevated
Urinalysis			
Color	Brown	Yellow, straw-colored	Abnormal
Appearance	Cloudy	Yellow, straw-colored	Abnormal
Protein	100	Negative, mg/dL	Abnormal
Blood	Large	Negative	Abnormal
Nitrite	Positive	Negative	Abnormal
Bacteria	Many	None, hpf	Abnormal
Urobilinogen	1.0	Negative, mg/dL	Abnormal
RBCs	>50	0 - 3 hpf	Abnormal

**Figure 1:**
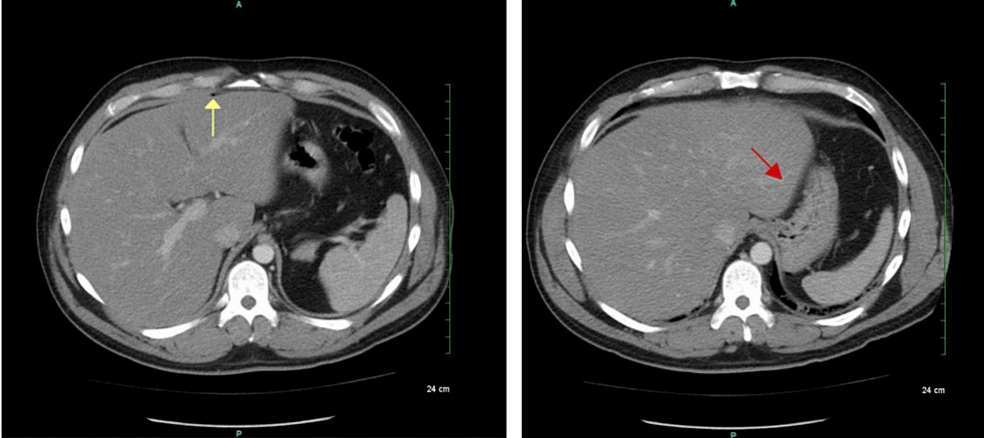
Computed tomography (CT) with intravenous (IV) contrast demonstrating multiple pockets of free air in the midline anterior abdominal wall and peritoneal cavity (yellow arrow). Air pocket tracking toward the liver margin with mesenteric induration and thickening (red arrow)

Computed tomography of the abdomen and pelvis with intravenous (IV) contrast demonstrated pockets of free air in the midline anterior abdominal wall and peritoneal cavity. (Figure 1). 

**Figure 2:**
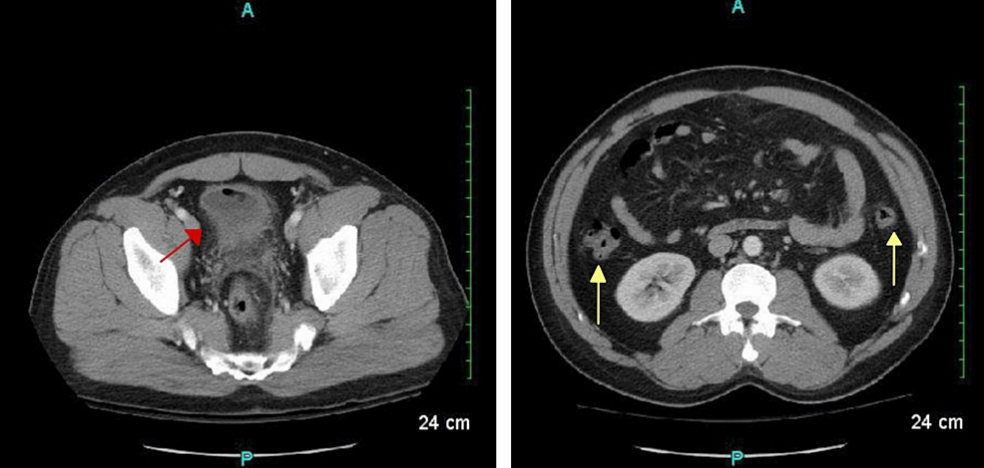
Computed tomography (CT) with intravenous (IV) contrast scan of sigmoid colon demonstrating (a) inflammatory changes (red arrow) and (b) colonic wall thickening and scattered diverticula (yellow arrows)

There was also an air pocket tracking toward the liver margin with mesenteric induration and thickening, suggesting possible perforation. There was no free fluid, phlegmon, or fluid collections representing an abscess. The sigmoid colon revealed inflammatory changes along with the presence of scattered diverticula and colonic wall thickening, making the diagnosis of acute diverticulitis likely (Figure 2).

**Figure 3:**
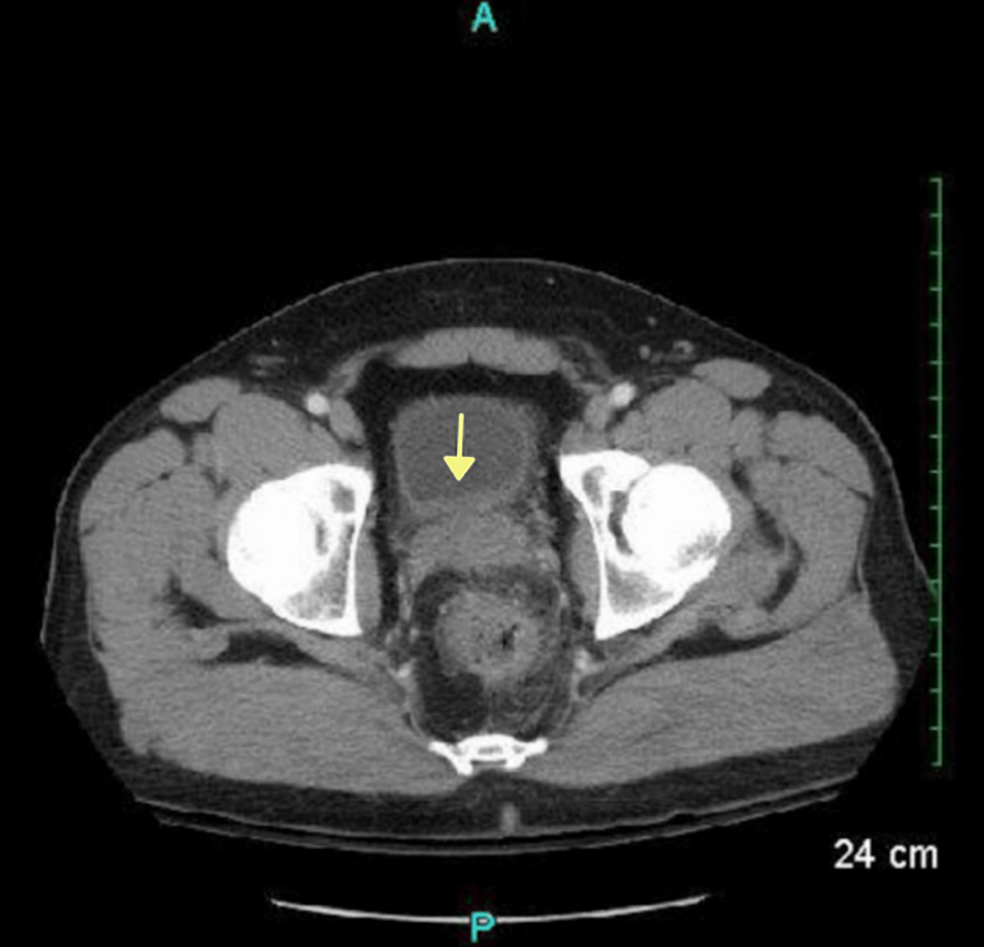
Computed tomography (CT) with intravenous (IV) contrast scan demonstrating pockets of air and irregular hyper-dense nodular material in the anterior bladder and diffuse urinary bladder wall thickening (yellow arrow)

Additional pockets of air and an irregular hyper-dense nodular material were found in the anterior bladder along with diffuse urinary bladder wall thickening, suggestive of a CVF (Figure 3). 

A diagnosis of sigmoid diverticulitis with a suspected CVF was made. The initial treatment plan was conservative management with bowel rest, continuous IV lactated Ringer's infusion, 5.0 mg metoprolol, 1.0 mg of IV ceftriaxone, and 500 mg IV metronidazole for adequate enteric microbial coverage. Over the next 48 hours, the patient clinically improved and was offered surgical intervention as there was a significant clinical concern for a CVF. He completed a mechanical and antibiotic bowel preparation and the next morning (admission day five), he underwent laparoscopic sigmoid colon resection with an interrogation of the CVF and primary anastomosis. 

Intraoperatively, there was no abscess, purulent fluid, or peritoneal contamination. There were inflammatory changes along the anterior sigmoid colon, attaching anteriorly to the dome of the bladder. After bluntly dissecting the colon from the bladder, the instillation of methylene blue through the Foley catheter did not identify a leak, and no CVF was found. The descending colon was healthy and viable. The diseased section of the sigmoid colon with the diverticulosis was resected and sent to pathology. Given the absence of peritoneal contamination and the completion of a mechanical and antibiotic bowel prep, a primary anastomosis was performed. A leak check with the proctoscope was negative for any bubbles or leaks. A Jackson-Pratt (JP) drain was placed into the pelvis. The procedure was tolerated well without any complications, and the patient was extubated. The patient was then transferred to the Post Anesthesia Care Unit (PACU) in stable condition.

**Figure 4:**
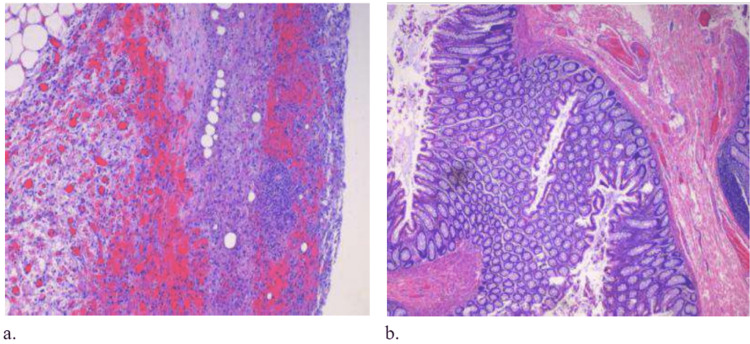
Hematoxylin and eosin (H&E) stained colonic tissue, demonstrating (a) reactive changes and (b) focal chronic inflammation.

The pathology report confirmed the presence of diverticula with focal chronic inflammation, fibrosis, focal hemorrhage, and reactive changes (Figure 4). No perforation was seen.

The patient’s postoperative course was uncomplicated. He remained afebrile throughout his hospital stay and was discharged on postoperative day two with a Foley catheter in place at the recommendation of the urologist. The patient followed up in the outpatient clinic two weeks post-procedure, on postoperative day eight. His incision sites were clean and dry and without any discharge. The Foley catheter revealed a clear urine output and was removed along with the JP drain. He appeared well and had no acute complaints. He was advised to follow up in six months for a colonoscopy to screen for occult malignancy.

## Discussion

Colonic diverticula are mucosal herniations in the muscle layer through weak points of entry of mesenteric blood vessels through the colonic wall [11]. A true diverticulum is defined by herniation through all three muscle layers: the mucosa, submucosa, and muscularis externa. An example is the congenital malformation known as Meckel’s diverticulum [11,12]. False diverticulum, on the other hand, includes herniation of only the mucosa and submucosa through a weakness in the muscularis externa [11], an effect that is seen with the majority of colonic diverticula [11,12]. 

Diverticular disease has been dubbed the deficiency disease of Western civilization [13,14]. The etiology and its link with a high-fiber diet were first hypothesized by Burkitt in 1971, who suggested that a high-fiber diet would result in a shorter transit time through the colon and would be beneficial for patients with diverticulitis [13,15]. Whiteway and colleagues presented an alternative explanation and reported that since the diverticula are present in two-thirds of the intestine between the mesenteric and antimesenteric taeniae, its presence results in the distinctive shortening of the muscle layer and an accordion-like defect termed the “concertina” effect [16]. They reported that compared to healthy controls, Taenia coli of those with diverticular disease have an elastin content that is increased by more than 200%. Eastwood and colleagues reported that collagen cross-linking is aberrant in individuals with diverticular disease, a pronounced effect seen after the age of 40, which is responsible for the peak incidence in this age demographic [17]. It was also hypothesized that an increase in collagen cross-linking decreases compliance which leads to tissue stiffness and susceptibility to tears with increased luminal pressures exerted by constipation, a classic risk factor.

Diverticulitis has varying presentations depending on the geographic region. In Western societies, the majority (65%) of colonic diverticula are located in the left sigmoid colon, which is in contrast to Asian countries, where it most commonly affects the right colon [18]. The remaining 35% of diverticula can be present in other segments of the colon. In up to 10% of patients, the diverticula are not localized and may be scattered throughout the colon [18]. In a Japanese study consisting of 28,192 patients, 48% of diverticula detected by colonoscopy were right-sided [19]. These results were later confirmed by another study of the Asian population, in which the prevalence of right-sided colonic diverticulosis was reported to be approximately 13 to 25% [3,20]. Notably, the prevalence varies between different age groups. The prevalence increases significantly with age which is reflected by the 50-70% prevalence rate in individuals over the age of 80, in contrast to the 10% prevalence rate of individuals under 40 [19].

The incidence of diverticulitis has been on the rise in recent years, particularly among individuals under 40 [15]. A study of diverticulitis hospitalization rates revealed that admission rates increased significantly in patients under 45, whereas the rates remained unchanged in patients over 65. The study was conducted in the United States and shows an emerging trend that is concerning, given that historically diverticular disease was mainly prevalent in older individuals [15]. 

Diverticulitis in younger patients has been shown to affect predominantly males when compared to its classical gender-neutral presentation in patients 50-70 years of age [3,18]. In addition, it has a more aggressive disease course with higher risks of complications and recurrence rates [19]. In younger patients, the clinical presentation of diverticulitis may be atypical and include rare symptoms. In one case report, a 20-year-old male patient described epigastric abdominal pain radiating to the lower abdominal quadrant as the presentation of acute diverticulosis [19]. Our case follows this emerging pattern of rare clinical presentation in younger patients.

Most cases of diverticulosis are asymptomatic. When they are symptomatic, patients present with localized LLQ pain. This symptomatic presentation reflects the underlying anatomic defect and the inflammatory process seen in the left-sided sigmoid colon [18]. Atypical presentations include RLQ abdominal pain, seen more commonly in Asian patients, and diffuse pain throughout the entire abdomen. Typically, the pain associated with diverticulitis can be constant or intermittent in nature. It is often associated with nausea and vomiting and is relieved by passing flatus or having a bowel movement. The presence of fever and leukocytosis reflects the progression of diverticulosis to diverticulitis, an inflammatory process [18], and can be suggestive of complications such as an abscess or perforation [3]. Another common complication is sympathetic cystitis which presents with dysuria, urinary frequency, and urgency. Symptoms such as pneumaturia and fecaluria reflect the development of a CVF, an abnormal connection between the bowel and the bladder [3]. Increased age, obesity, and low-fiber diets are known risk factors that may become apparent with thorough clinical history taking [6]. 

This patient presented with atypical findings that would not be expected in a typical diverticulitis patient. As mentioned previously, the geographic regions of the world depict pain in distinctive patterns. Literature reports that both LLQ and RLQ pain can be seen depending on the geographic region under discussion [18]. However, our patient presented with focal epigastric tenderness and non-tender lower and lateral quadrants, findings which, to the best of the authors’ knowledge, have not been reported in the literature and warrant a potential avenue for future research in this age group. Additionally, many classic risk factors such as advanced age, smoking, and a history of constipation were not present in this patient. Despite marked leukocytosis, the patient remained afebrile throughout his hospital stay. Moreover, although the physical examination and radiological findings were positive for the presence of pneumaturia and fecaluria, administration of the methylene blue dye failed to reveal a CVF intraoperatively. One possibility is that the fistula could have healed and closed by the time of surgical exploration, another variable clinical presentation specific to this age group. 

One limitation of this study is the nature of the study itself. Since this case report only follows one individual's presentation, we cannot conclusively say that his unique presentation is representative of his cohort or is generalizable to the population. Future studies need to focus on assessing the clinical presentation of diverticulitis in young Hispanic males to understand if there is indeed a distinctive pattern of disease presentation, related complications, and outcomes.

## Conclusions

The objective of this case is to make clinicians aware of atypical presentations of acute diverticulitis and guide the appropriate diagnostic workup for young patients presenting to the emergency departments with complaints of epigastric pain. We found that the location of the diverticular disease within the bowel is influenced by geographic region and associated dietary habits which has been supported by the literature. The existing literature also suggests an increase in the incidence of diverticular disease in younger age groups, however, the presentation has contradictory evidence for younger and older age groups. Diverticulitis presents clinically with focal pain in LLQ and RLQ; however, epigastric pain, as seen in our case, is not typically associated with diverticulitis. This calls into question if younger patients have more diversified and atypical presentations. Further research is needed to find an answer to this question and learn more about diverticulitis in patients under 40 years of age.

One limitation of this study is the nature of the study itself. Since this case only follows one individual's presentation, we cannot conclusively say that his unique presentation is representative of his cohort or is generalizable to the population. Future studies need to focus on assessing the clinical presentation of diverticulitis in young males to understand if there is indeed a distinctive pattern of disease presentation, related complications, and outcomes. We highlight the dire need for well-designed long-term future studies to investigate the age and racial/ethnic influence on the diverticulitis disease course. Moreover, we highlight the importance of monitoring by primary care physicians of low-income, uninsured, minority individuals to lower the cost burden associated with hospital admissions and in lowering the incidence of preventable emergency surgical procedures.
